# Ellagic acid microspheres restrict the growth of *Babesia* and *Theileria in vitro* and *Babesia microti in vivo*

**DOI:** 10.1186/s13071-019-3520-x

**Published:** 2019-05-28

**Authors:** Amani Magdy Beshbishy, Gaber El-Saber Batiha, Naoaki Yokoyama, Ikuo Igarashi

**Affiliations:** 1 National Research Center for Protozoan Diseases, Obihiro University of Agriculture and Veterinary Medicine, Nishi 2-13 Inada-cho, Obihiro, Hokkaido 080-8555 Japan; 2 Department of Pharmacology and Therapeutics, Faculty of Veterinary Medicine, Damanhour University, Damanhour, 22511 El-Beheira Egypt

**Keywords:** Ellagic acid, β-cyclodextrin ellagic acid, APSP EA, Nanoparticles, *Babesia*, *Theileria*

## Abstract

**Background:**

There are no effective vaccines against *Babesia* and *Theileria* parasites; therefore, therapy depends heavily on antiprotozoal drugs. Treatment options for piroplasmosis are limited; thus, the need for new antiprotozoal agents is becoming increasingly urgent. Ellagic acid (EA) is a polyphenol found in various plant products and has antioxidant, antibacterial and effective antimalarial activity *in vitro* and *in vivo* without toxicity. The present study documents the efficacy of EA and EA-loaded nanoparticles (EA-NPs) on the growth of *Babesia* and *Theileria*.

**Methods:**

In this study, the inhibitory effect of EA, β-cyclodextrin ellagic acid (β-CD EA) and antisolvent precipitation with a syringe pump prepared ellagic acid (APSP EA) was evaluated on four *Babesia* species and *Theileria equi in vitro*, and on the multiplication of *B. microti* in mice. The cytotoxicity assay was tested on Madin-Darby bovine kidney (MDBK), mouse embryonic fibroblast (NIH/3T3) and human foreskin fibroblast (HFF) cell lines.

**Results:**

The half-maximal inhibitory concentration (IC_50_) values of EA and β-CD EA on *B. bovis*, *B. bigemina*, *B. divergens*, *B. caballi* and *T. equi* were 9.58 ± 1.47, 7.87 ± 5.8, 5.41 ± 2.8, 3.29 ± 0.42 and 7.46 ± 0.6 µM and 8.8 ± 0.53, 18.9 ± 0.025, 11 ± 0.37, 4.4 ± 0.6 and 9.1 ± 1.72 µM, respectively. The IC_50_ values of APSP EA on *B. bovis*, *B. bigemina*, *B. divergens*, *B. caballi* and *T. equi* were 4.2 ± 0.42, 9.6 ± 0.6, 2.6 ± 1.47, 0.92 ± 5.8 and 7.3 ± 0.54 µM, respectively. A toxicity assay showed that EA, β-CD EA and APSP EA affected the viability of cells with a half-maximal effective concentration (EC_50_) higher than 800 µM. In the experiments on mice, APSP EA at a concentration of 70 mg/kg reduced the peak parasitemia of *B*. *microti* by 68.1%. Furthermore, the APSP EA-atovaquone (AQ) combination showed a higher chemotherapeutic effect than that of APSP EA monotherapy.

**Conclusions:**

To our knowledge, this is the first study to demonstrate the *in vitro* and *in vivo* antibabesial action of EA-NPs and thus supports the use of nanoparticles as an alternative antiparasitic agent.

**Electronic supplementary material:**

The online version of this article (10.1186/s13071-019-3520-x) contains supplementary material, which is available to authorized users.

## Background

*Babesia* and *Theileria* are the most common blood-borne parasites of mammals after the trypanosomes. They are the etiological agents of babesiosis and theileriosis, the first recognized vector-borne diseases that can infect a wide range of mammals, including humans [1]. Chemical therapy against piroplasmosis in the livestock industries remains insufficient. Although diminazene aceturate (DA) and imidocarb dipropionate showed several challenges (such as the development of toxicity, drug-resistant parasites, drug residues and withdrawal issues) that hinder the use of these drugs in many countries [2], they are still the only options for the treatment of bovine and equine piroplasmosis [3]. Moreover, they are not approved for human medicine. The preferable treatment of babesiosis in humans is the combination of atovaquone (AQ) with azithromycin due to their low side effects [4]. Guler et al. [5] reported that *Plasmodium falciparum* rapidly developed resistance to AQ when used as a single drug. Another report showed the relapse of *Babesia gibsoni* due to the change of amino acid in the mitochondrial cytochrome B that led to a reduction in the efficacy of AQ [6]. It is noteworthy that the discovery of new molecules creates a pool of potential compounds for the selection of drugs to advance into clinical trials.

Ellagic acid (EA; C_14_H_6_O_8_) is a naturally occurring phenolic constituent that is contained in ellagitannins in grapes, strawberries, black currants, raspberries, green tea and many herbal plants [7]. EA has potent preventive and therapeutic effects against several types of cancers, including colon cancer, breast cancer, prostate cancer, skin cancer, esophageal cancer and osteogenic sarcoma [8]. Additionally, it can stimulate apoptosis and completely inhibit the proliferation of human pancreatic adenocarcinoma cell lines MIA PaCa-2 and PANC-1 through decreasing nuclear factor-kappa B (NF-κB) activity, activating the mitochondrial death pathway, which is associated with the loss of mitochondrial membrane potential, cytochrome C release and caspase-3 activation [9]. EA is a naturally occurring broad-spectrum antioxidant that acts by direct scavenging of nitrogen reactive species and ROS, including hydroxyl radicals, peroxyl radicals, NO_2_ radicals and peroxynitrite [10]. EA reportedly possesses anti-inflammatory properties through interaction with known cyclooxygenase inhibitors [11]. Recently, the antimalarial properties of EA were evaluated, and the results obtained have shown high activity *in vitro* against all *P. falciparum* strains. Additionally, it was active *in vivo* against *P. vinckei petteri* in suppressive, curative and prophylactic murine tests, without any toxicity [7, 12]. Aminu et al. [13] reported the *in vitro*, *in silico* antisialidase activities and the *in vivo* antitrypanosomal potentials of EA. Moreover, Alves et al. [14] revealed the antileishmanial activity of EA on promastigote forms of *Leishmania major*.

In spite of the above-mentioned important medicinal properties of EA, it still has poor bioavailability, poor aqueous solubility, instability and is easily oxidized by heat. Furthermore, EA has a short plasma half-life because its oral administration leads to a lactone ring opening by the intestinal microorganism and is rapidly eliminated from the body, which are the main causes of its low bioavailability [15]. Therefore, the preparation of slow-release ellagic acid microspheres could solve these problems [16]. Nanoparticles (NPs) are considered to be the most promising delivery system for compounds with low bioavailability. Many well-known carriers have been developed as effective drug delivery systems, including liposomes, biodegradable polymeric nanoparticles, hydrogels and cyclodextrin [15, 17]. These nanocarriers are able to protect encapsulated drugs from gastrointestinal degradation and first-pass metabolism. Moreover, nanoparticles are able to maintain the drug release in the plasma for a longer time period, thereby reducing the frequency of administration [18].

In the present study, two types of EA-loaded nanoparticles (EA-NPs) were prepared: β-cyclodextrin ellagic acid (β-CD EA) and antisolvent precipitation using a syringe pump prepared ellagic acid (APSP EA). β-Cyclodextrin (β-CD) is a cyclodextrin cyclic oligosaccharide produced by cyclodextrin glucose transferase. The formation of inclusion compounds is one of the most important features of β-CD [16]. β-CD is hydrophobic inside and hydrophilic outside; thus, it can form complexes with hydrophobic compounds and enhance the solubility, bioavailability and permeability of such compounds through mucosal tissues [19]. For the antisolvent precipitation of poorly water-soluble drugs, the drug is first dissolved in a solvent and then rapidly mixed with a solvent-miscible antisolvent (water) [20]. The APSP has several advantages in being a simple pumping, stirring, filtering system, and water was used as an antisolvent. Despite many pharmacologic investigations, there have been no reports on the antibabesial activity of EA and EA-NPs. Therefore, this study aimed to evaluate the effects of EA and EA-NPs against the growth of *Babesia bovis*, *Babesia bigemina*, *Babesia divergens*, *Babesia caballi* and *Theileria equi in vitro* and their chemotherapeutic potential on *Babesia microti* in mice. Furthermore, we investigated the effect of combination between EA and EA-NPs with the current babesiocidal drugs such as DA, AQ and clofazimine (CF) on the *in vitro* growth of *B. bovis*, *B. bigemina*, *B. divergens*, *B. caballi* and *T. equi*, and their chemotherapeutic activities against *B. microti* in mice.

## Methods

### Chemicals and reagents

**Fig. 1:**
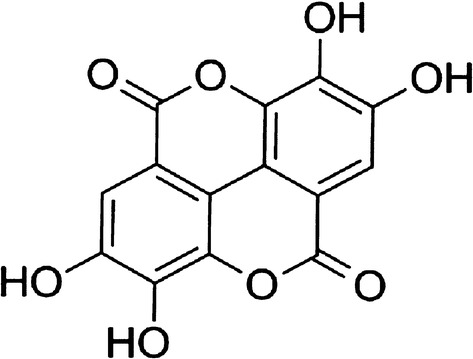
Chemical structure of ellagic acid

EA (Fig. 1), DA, CF and AQ powders (Sigma-Aldrich, Tokyo, Japan) were prepared in dimethyl sulfoxide (DMSO) in stock solutions of 10 Mm, which was stored at − 30 °C until examination of its babesiocidal effects. DA, CF and AQ were used as comparator drugs *in vitro* and used in combination with EA or EA microspheres either *in vitro* or *in vivo*. β-CD (Sigma Chemical Co., St. Louis, MO, USA) and 99.5% ethanol (Chameleon Reagent, Osaka, Japan) were used for the preparation of EA microspheres. SYBR Green I nucleic acid stain (SGI, 10,000×; Lonza, Alpharetta, GA, USA) was purchased, wrapped in aluminum foil for protection from direct light, and stored at − 30 °C. A lysis buffer containing Tris-HCl (130 mM at pH 7.5), EDTA (10 mM), saponin (0.016%; w/v) and Triton X-100 (1.6%; v/v) was prepared, filtered through 0.22 µm of polyether sulfone, and stored at 4 °C.

### Preparation of ellagic acid microspheres

#### Preparation of β-CD-ellagic acid

The assay was conducted in accordance with the protocol described previously with slight modification [16]. Briefly, for saturated aqueous solution preparation, 5 g of β-CD was dissolved in 200 ml of double-distilled water (DDW). At the same time, 1.25 g of EA was dissolved in 10 ml of ethanol, followed by slowly dripping off the EA solution into the β-CD saturated solution. The mixture was stirred using magnetic stirring at room temperature for 2 h, placed in a refrigerator overnight, and then vacuum dried for several hours. β-CD EA microspheres were characterized by UV/Vis spectrophotometry and inductively coupled plasma optical emission spectrometry (ICP-OES) [21].

#### Antisolvent precipitation with a syringe pump (APSP) method

EA was prepared in ethanol at the predetermined concentration of 10 mg/ml. The syringe was filled with 20 ml of the prepared solution and connected with a syringe pump. The drug solution was quickly injected at a fixed flow rate (2–10 ml/min) into the deionized water (antisolvent) with a ratio of 1:10 (v/v) and stirred with a magnetic stirrer. The EA nanoparticles that formed were filtered and vacuum dried. APSP EA nanoparticles were characterized by the use of UV/Vis spectrophotometry and inductively coupled plasma optical emission spectrometry (ICP-OES). Scanning electron microscopy (SEM; JSM-7500F, JEOL, Tokyo, Japan) was used to evaluate surface morphology of the prepared particles. Prior to examination, samples were sputter coated with platinum using an auto fine coater (JFC-1600, JEOL) to render them electrically conductive [20].

### Parasites and mice

A microaerophilic stationary-phase culture system was used to maintain *Babesia* parasite cultures for a long time [22, 23]. Briefly, *B. caballi* (USDA strain) was grown using equine red blood cells (RBCs) in GIT medium supplemented with 40% equine serum. *Theileria equi* (USDA strain) was grown in equine RBCs in Medium 199 (M199; Sigma-Aldrich, Tokyo) supplemented with 40% equine serum, and hypoxanthine (MP Biomedicals, Santa Ana, CA, USA) at a final concentration of 13.6 μg/ml was used as a vital supplement. *Babesia bovis* (Texas strain) and *B. bigemina* (Argentina strain) were grown in bovine RBCs in M199 medium supplemented with 40% bovine serum [24]. Meanwhile, *B. divergens* (Germany strain) was grown in bovine RBCs in medium RPMI 1640 (Sigma-Aldrich, Tokyo) supplemented with 40% bovine serum [25]. All media included 60 U/ml penicillin G, 60 μg/ml streptomycin and 0.15 μg/ml amphotericin B (Sigma-Aldrich, St. Louis, MO, USA) to prevent bacterial contamination. The cultures were incubated at 37 °C in a humidified chamber with an atmosphere of 5% CO_2_, 5% O_2_ and 90% N_2_. *Babesia microti* (Munich strain) was recovered from − 80 °C stock, intraperitoneally injected in two 8-week old female BALB/c mice (Clea Japan, Tokyo, Japan) [26], and the parasitemia was monitored every 2 days. The mice were euthanized using an anesthesia system containing isoflurane after parasitemia reached approximately 30%, and blood was collected by cardiac puncture to initiate the *in vivo* experiment [27]. The animal experiment was conducted in accordance with the Regulations for Animal Experiments of Obihiro University of Agriculture and Veterinary Medicine, Japan (animal experiment accession number: 290168).

### Evaluation of the effect of EA, β-CD EA and APSP EA on cattle and horse RBCs

Bovine and equine RBCs were incubated with 10, 100 and 200 μM EA, β-CD EA and APSP EA for 3 h at 37 °C to be used for the parasite subculture. The RBCs were washed three times with phosphate-buffered saline (PBS) and mixed with *B. bovis-* and *T. equi*-parasitized RBCs to obtain 1% parasitemia. Afterwards, in a 24-well plate, 100 μl of infected RBCs (iRBCs) was added to 900 μl of complete medium. Untreated RBCs were used as a control. Giemsa-stained blood smears were prepared daily to observe any side effects as a result of the pretreatment.

### Growth inhibition assay of EA, β-CD EA and APSP EA and a combination with DA, CF and AQ *in vitro*

Assays were performed in accordance with the previously described protocol [28]. Briefly, *B. bovis*, *B. bigemina*, *B. divergens*, *B. caballi* and *T. equi* cultures were harvested and adjusted to 1% parasitemia with fresh RBCs to start the inhibition assay. The assays were carried out using 96-well microtiter plates where only the 60 inner wells were used, while the peripheral wells were filled with sterile distillate water to reduce evaporation during incubation. In five 96-well plates, a volume of 2.5 µl each of *B. bigemina-* and *B. bovis*-iRBCs and 5 µl each of *B. divergens*-, *B. caballi*- and *T. equi*-iRBCs were added to each well in triplicate and mixed with a culture medium containing the drug concentrations to a total volume of 100 µl. Various concentrations of EA, β-CD EA, APSP EA, DA, CF and AQ were dissolved in a culture medium using two-fold dilution. Wells containing DMSO at a final concentration of 0.4% and iRBCs were used as a positive control, while wells with non-infected RBCs and culture medium were used as a negative control. Additionally, a drug combination assay was performed in parallel with the single drug assay at a constant ratio of 1:1, as described previously [29]. Two-drug combinations at 5 selected concentrations of 0.25 × IC_50_, 0.5 × IC_50_, IC_50_, 2 × IC_50_ and 4 × IC_50_ were added to wells containing iRBCs in duplicate in the same 96-well plates with a single-drug inhibition assay. The plates were incubated in a humidified incubator with 5% CO_2_, 5% O_2_ and 90% N_2_ for 4 days without changing the medium. On day 4, 100 µl of a lysis buffer containing 2 × SG1 was added to each well. The plates were wrapped with aluminum foil to avoid direct light exposure and incubated at room temperature. After 6 h, fluorescence values were measured using a fluorescence spectrophotometer (Fluoroskan Ascent; Thermo LabSystems, Oceanside, CA, USA) with excitation and emission wavelengths of 485 and 518 nm, respectively. The fluorescence data were subtracted from the negative control and used to calculate the IC_50_. To calculate the degree of association, the growth-inhibition values obtained were entered into CompuSyn software, based on the combination index (CI) values. The CI values of the drug combinations were determined using the formula [(1 × IC_50_) + (2 × IC_75_) + (3 × IC_90_) + (4 × IC_95_)]/10, and the results were interpreted using the reference combination index scale of < 0.90 (synergism), 0.90–1.10 (additive) and > 1.10 (antagonism) developed previously [29]. The experiments were repeated three times.

### Cell cultures

Madin-Darby bovine kidney (MDBK), mouse embryonic fibroblast (NIH/3T3) and human foreskin fibroblast (HFF) cell lines were maintained for a long time using a 37 °C humidified incubator with 5% CO_2_. The MDBK cell line was maintained in 75 cm^2^ culture flasks with Minimum Essential Medium Eagle (MEM; Gibco, Grand Island, NY, USA), while Dulbecco’s Modified Eagle’s Medium (DMEM; Gibco) was used for the cultivation of NIH/3T3 and HFF cell lines. Each medium was supplemented with 10% fetal bovine serum, 0.5% penicillin/streptomycin (Gibco), and an additional 1% glutamine. Every 3 to 4 days, the medium was changed, and the cells were incubated until approximately 80% confluent. The cells were free from mycoplasma contamination after being checked by staining with 4,6-diamidino-2-phenylindole dihydrochloride (DAPI; Sigma-Aldrich, St. Louis). The cell detachment from the culture flask was done using TrypLE^TM^ Express (Gibco) after washing two times with Dulbecco’s phosphate-buffered saline (DPBS). Subsequently, viable cells were counted using a Neubauer Improved C-Chip (NanoEnTek Inc., Seoul, Korea) after staining with 0.4% trypan blue solution.

### Cytotoxicity assay of EA, β-CD EA, APSP EA, DA, CF and AQ on MDBK, NIH/3T3 and HFF cell lines

The drug-exposure viability assay was performed in accordance with the recommendation for the Cell Counting Kit-8 (CCK-8; Dojindo, Kumamoto, Japan). Briefly, in a 96-well plate, 100 µl of cells at a density of 5 × 10^4^ cells/ml were seeded per well and allowed to attach to the plate for 24 h at 37 °C in a humidified incubator with 5% CO_2_. Ten microliters of drug diluted two-fold were added to each well to a final concentration of 0.781 to 800 µM in triplicate. Wells with only a culture medium were used as blanks, while wells containing cells and a medium with 0.5% DMSO were used as positive controls. Drug exposure lasted for 24 h. After 24 h, 10 µl of CCK-8 was added, and the plate was further incubated for 3 h. Absorbance was measured at 450 nm using a microplate reader.

### *In vivo* growth inhibition and drug combination assays in mice

*In vivo* growth inhibition assays of EA, β-CD EA and APSP EA were performed in *B. microti*-infected BALB/c mice in accordance with the previous protocol [28] in two separate trials. Briefly, 40 female 8-week-old BALB/c mice were divided into 8 groups, each consisting of 5 mice. The mice in groups 2–8 were intraperitoneally (i.p.) injected with 0.5 ml of inoculum (1 × 10^7^ *B. microti-*iRBCs). Group 1 was left uninfected and untreated as a negative control. When the average parasitemia in all mice reached 1%, drug treatment was initiated for 5 consecutive days. Group 2 was treated with 5% DMSO in DDW i.p. injected as a positive control. DA at a concentration of 25 mg/kg body weight (BW) was administrated i.p. to the third group as a reference drug control. Groups 4–8 received an i.p. injection of 30 mg/kg BW of EA, an i.p. injection of 140 mg/kg BW of β-CD EA, an oral administration of 200 mg/kg BW of β-CD EA, an i.p. injection of 70 mg/kg BW of APSP EA, and an oral administration of 120 mg/kg BW of APSP EA, respectively.

To validate the efficacy of the combinations of APSP EA with DA, CF and AQ *in vivo*, 40 female 8-week-old BALB/c mice were divided into 8 groups, each consisting of 5 mice, which were intraperitoneally inoculated with 1 × 10^7^ *B. microti*-iRBCs in two separate trials. Another group was kept uninfected and untreated as a negative control. Group 2 was treated with 5% DMSO in DDW i.p. as a positive control. Groups 3–6 were given an i.p. injection of 25 mg/kg BW of DA, an oral administration of 20 mg/kg BW of CF, an oral administration of 20 mg/kg BW of AQ, and an i.p. injection of 70 mg/kg BW of APSP EA, respectively. Groups 7–9 were treated with combinations of APSP EA + DA (35 + 12.5 mg/kg BW), APSP EA + CF (35 + 10 mg/kg BW), and APSP EA + AQ (35 + 10 mg/kg BW), respectively, using the same route of each drug as before. The drug treatment was conducted for 5 days, and the parasitemia was estimated from Giemsa-stained blood smears using microscopy in approximately 5000 RBCs. Moreover, the hematology profiles including RBCs, hemoglobin (HGB) and hematocrit (HCT) counts were measured from 10 µl of mouse blood using an automatic hematology analyzer (Celltac α MEK-6450; Nihon Kohden, Tokyo, Japan). The rate of parasitemia and hematology profiles were monitored every 2 and 4 days, respectively, until day 60. On days 42 and 56, blood was collected for PCR detection of parasites.

### Genomic DNA extraction and PCR detection of *B. microti*

A QIAamp DNA Blood Mini Kit (Qiagen, Tokyo, Japan) was used to extract genomic DNA from the blood. A nested PCR (nPCR) targeting the *B. microti* small subunit rRNA (ss-rRNA) gene was conducted as previously described [24]. Briefly, PCR amplifications were performed in a 10 µl reaction mixture containing 0.5 µM of each primer, 2 µl of 5× SuperFi™ buffer, 0.2 mM dNTP mix, 0.1 µl of Platinum SuperFi™ DNA polymerase (Thermo Fisher Scientific, Tokyo, Japan), 1 µl of DNA template and 4.9 µl of DDW. The cycling conditions were: 94 °C for 30 s, 53 °C for 30 s and 72 °C for 30 s as denaturation, annealing and extension steps for 35 cycles, respectively, using the forward (5′-CTT AGT ATA AGC TTT TAT ACA GC-3′) and reverse (5′-ATA GGT CAG AAA CTT GAA TGA TAC A-3′) primers. Afterward, 1 µl of DNA template from the first PCR amplification was used as the template for the nPCR assays under the same cycling conditions, using the forward (5′-GTT ATA GTT TAT TTG ATG TTC GTT T-3′) and reverse (5′-AAG CCA TGC GAT TCG CTA AT-3′) primers. The PCR products were stained with ethidium bromide and visualized under the UV transilluminator after resolution by electrophoresis in 1.5% agarose gel. Bands with an expected size of 154 bp were considered positive.

### Statistical analysis

The IC_50_s of EA, β-CD EA, APSP EA, DA, CF and AQ were calculated from the percentage of inhibition of the *in vitro* growth of all tested species using non-linear regression (curve fit), available in GraphPad Prism (GraphPad Software Inc., La Jolla, CA, USA). The differences among groups in the *B. microti*-infected mouse model regarding the parasitemia and hematology profiles were analyzed using Student’s t-test, available in GraphPad Prism software. The difference was considered significant if *P* < 0.05.

## Results

### EA nanoparticle synthesis and characterization

**Fig. 2:**
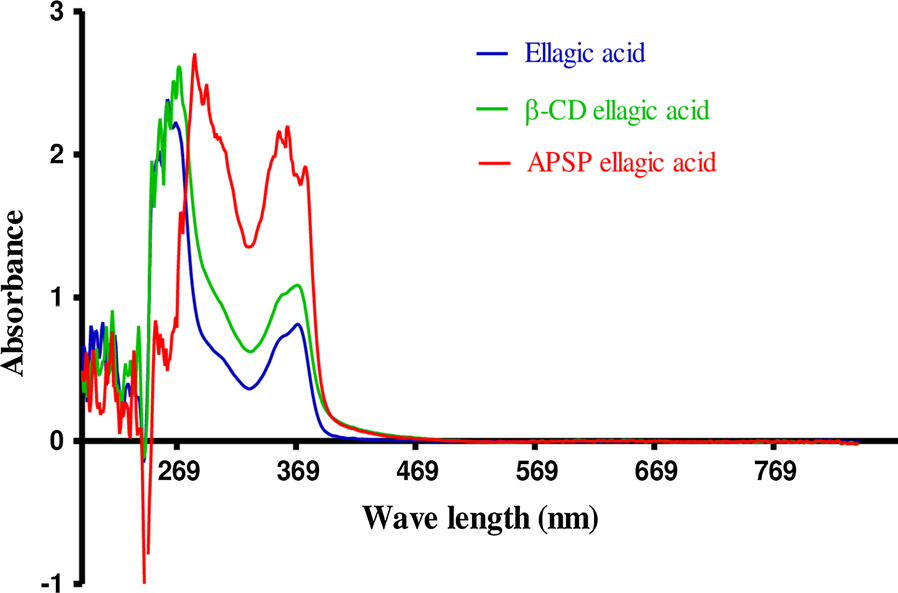
UV/Vis absorbance spectra of nanoparticles (150–800 nm)

**Fig. 3:**
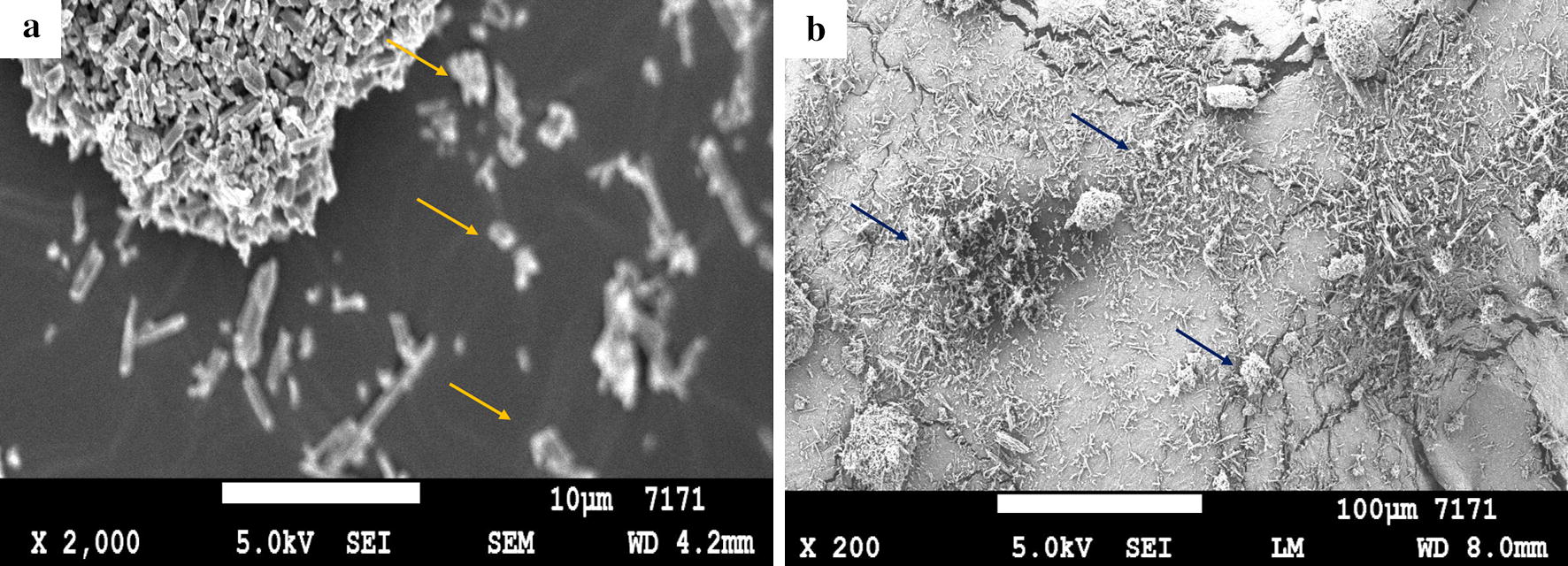
**a**, **b** Scanning electron microscope (SEM) image of APSP EA nanoparticles. The arrows show the needle-shaped or rod-like crystal structure of APSP EA nanoparticles. *Scale-bars*: **a**, 10 µm; **b**, 100 µm

The UV/Vis spectral characterization of EA-NPs showed strong absorption at 255 and 375 nm (Fig. 2) that is compatible with Bulani et al. [30], which confirms the successful synthesis of the nanoparticles. The SEM images showed that APSP EA particles appear to have a needle-shaped or rod-like crystal structure and exhibit good dispersion (Fig. 3a, b). This finding is consistent with Kakran et al. [20], who revealed that the curcumin nanoparticles prepared by APSP method showed significant changes in the curcumin crystalline habitus. Modification in the shape of crystals and aspect is suggested as a proof of production of nanoparticles with higher solubility and a higher rate of dissolution [20].

### The growth-inhibitory effect of EA, β-CD EA and APSP EA against *Babesia* and *Theileria in vitro*

Growth-inhibitory assays were conducted on five species: *B. bovis*, *B. bigemina*, *B. divergens*, *B. caballi* and *T. equi*. EA, β-CD EA and APSP EA inhibited the multiplication and growth of all species tested in a dose-dependent manner. EA significantly inhibited the growth of *B. bovis*, *B. bigemina*, *B. caballi* and *T. equi* (t-test: *t*_(9)_ = 2.993, *P* = 0.001) at 0.078, 0.078, 0.125 and 0.125 µM, respectively, and *B. divergens* (t-test: *t*_(9)_ = 3.670, *P* = 0.005) at 0.125 µM (Additional file 1: Figure S1a). APSP EA significantly inhibited the growth of *B. bovis* and *B. caballi* (t-test: *t*_(7)_ = 3.406, *P* = 0.003) at 0.313 µM and significantly inhibited the growth of *B. bigemina*, *B. divergens* and *T. equi* (t-test: *t*_(7)_ = 3.406, *P* = 0.003) at 0.625 µM (Additional file 1: Figure S1b). The *in vitro* growth of *Babesia* parasites tested was significantly inhibited (t-test: *t*_(7)_ = 3.428, *P* = 0.001 for *B. bigemina* and *B. caballi*; *t*_(7)_ = 2.398, *P* = 0.003 for *B. bovis* and *B. divergens*) by 0.625 μM treatment of β-CD EA, while the *in vitro* growth of *T. equi* was significantly inhibited (t-test: *t*_(7)_ = 2.426, *P* = 0.005) by treatment with 0.625 µM β-CD EA (Additional file 1: Figure S1c). The IC_50_ values of EA and β-CD EA on *B. bovis*, *B. bigemina*, *B. divergens*, *B. caballi* and *T. equi* were 9.58 ± 1.47, 7.87 ± 5.8, 5.41 ± 2.8, 3.29 ± 0.42 and 7.46 ± 0.6 µM and 8.8 ± 0.53, 18.9 ± 0.025, 11 ± 0.37, 4.4 ± 0.6 and 9.1 ± 1.72 µM, respectively. The IC_50_ values of APSP EA on *B. bovis*, *B. bigemina*, *B. divergens*, *B. caballi* and *T. equi* were 4.2 ± 0.42, 9.6 ± 0.6, 2.6 ± 1.47, 0.92 ± 5.8 and 7.3 ± 0.54 µM, respectively (Table 1). In this study, DA showed IC_50_ values of 0.35 ± 0.06, 0.68 ± 0.09, 0.43 ± 0.05, 0.022 ± 0.0002 and 0.71 ± 0.05 µM against *B. bovis*, *B. bigemina*, *B. divergens*, *B. caballi* and *T. equi*, respectively. AQ showed IC_50_ values of 0.039 ± 0.002, 0.701 ± 0.04, 0.038 ± 0.002, 0.102 ± 0.0141 and 0.095 ± 0.0655 µM against *B. bovis*, *B. bigemina*, *B. divergens*, *B. caballi* and *T. equi*, respectively. CF showed IC_50_ values of 8.24 ± 1.7, 5.73 ± 1.9, 13.85 ± 4.3, 7.95 ± 1.8 and 2.88 ± 0.9 µM against *B. bovis*, *B. bigemina*, *B. divergens*, *B. caballi* and *T. equi*, respectively (Additional file 2: Table S1). The effectiveness of EA, β-CD EA and APSP EA were not influenced by the diluent since there was no significant difference in inhibition between wells containing DMSO and untreated wells. RBCs were precultivated with EA, β-CD EA and APSP EA to determine the direct effect on host RBCs. Bovine and equine RBCs were incubated with three different concentrations of EA, β-CD EA or APSP EA at 10, 100 and 200 µM for 3 h prior to the subculture of *B. bovis* and *T. equi*. The multiplication of *B. bovis* and *T. equi* did not significantly differ between EA-treated, β-CD EA-treated or APSP EA-treated RBCs and normal RBCs for either species (data not shown).

**Table 1:** The IC_50_ and selectivity index of EA, β-CD EA and APSP EA

Compound	Parasite	IC_50_ (µM)^a^	EC_50_ (µM)^b^			Selective indices^c^		
			MDBK	NIH/3T3	HFF	MDBK	NIH/3T3	HFF
EA	*B. bovis*	9.6 ± 1.5	> 800	> 800	> 800	> 83.3	> 83.3	> 83.3
	*B. bigemina*	7.9 ± 5.8				> 101.3	> 101.3	> 101.3
	*B. divergens*	5.4 ± 2.8				> 148.1	> 148.1	> 148.1
	*B. caballi*	3.3 ± 0.4				> 242.4	> 242.4	> 242.4
	*T. equi*	7.5 ± 0.6				> 106.7	> 106.7	> 106.7
	*P. falciparum*	330 nM^d^						
	*L. major*	9.8 μg/ml^e^						
β-CD EA	*B. bovis*	8.8 ± 0.53	> 800	> 800	> 800	> 90.9	> 90.9	> 90.9
	*B. bigemina*	18.9 ± 0.02				> 42.3	> 42.3	> 42.3
	*B. divergens*	11.0 ± 0.4				> 72.7	> 72.7	> 72.7
	*B. caballi*	4.4 ± 0.6				> 181.8	> 181.8	> 181.8
	*T. equi*	9.1 ± 1.7				> 87.9	> 87.9	> 87.9
APSP EA	*B. bovis*	4.2 ± 0.4	> 800	> 800	790 ± 5.4	> 190.5	> 190.5	188.1
	*B. bigemina*	9.6 ± 0.6				> 83.3	> 83.3	82.3
	*B. divergens*	2.6 ± 1.3				> 307.7	> 307.7	303.8
	*B. caballi*	0.9 ± 5.8				> 888.9	> 888.9	877.8
	*T. equi*	7.3 ± 0.5				> 109.6	> 109.6	108.2

^a^Half-maximal inhibition concentration of EA, β-CD EA and APSP EA on the *in vitro* culture of parasites. The value was determined from the dose-response curve using non-linear regression (curve fit analysis). The values are the means of experiments in triplicate

^b^Half-maximal effective concentration of EA, β-CD EA and APSP EA on cell lines. The values were determined from the dose-response curve using non-linear regression (curve fit analysis). The values are the means of experiments in triplicate

^c^Ratio of the EC_50_ of cell lines to the IC_50_ of each species. High numbers are favorable

^d^[7]

^e^[14]

*Abbreviations*: EA, ellagic acid; β-CD EA, β-cyclodextrin ellagic acid; APSP EA, antisolvent precipitation with a syringe pump prepared ellagic acid; MDBK, Madin–Darby bovine kidney; NIH/3T3, mouse embryonic fibroblast; HFF, human foreskin fibroblast

### The effects of EA, β-CD EA and APSP EA combined with DA, AQ and CF *in vitro*

Drug combinations were analyzed to determine whether the combined treatments are synergistic (give a greater effect), additive (similar effect) or antagonistic (reduce or block the effect). Five concentrations of EA, β-CD EA and APSP EA, as recommended in the Chou–Talalay method [29], were combined at a constant ratio (1:1) with DA, AQ and CF. The percentage of inhibition of the single drug and each combination was analyzed using CompuSyn software to generate the combination index (CI) values (Table 2). Combination treatments of EA-DA showed an additive effect against *B. bigemina* and a synergistic effect against *B. bovis*, *B. divergens*, *B. caballi* and *T. equi*. Combination treatments of EA-AQ showed a synergistic effect against *B. bovis*, *B. bigemina*, *B. divergens*, *B. caballi* and *T. equi*. Combination treatments of EA-CF showed a synergistic effect against *B. bovis* and *B. bigemina*, but an additive effect against *B. divergens*, *B. caballi* and *T. equi*. Combination treatments of β-CD EA-DA showed a synergistic effect against *B. caballi* but an additive effect against *B. bovis*, *B. bigemina*, *B. divergens* and *T. equi.* Combination treatments of β-CD EA-AQ showed a synergistic effect against all tested parasites. Combination treatments of β-CD EA-CF showed a synergistic effect against *B. bovis* and *B. divergens*, but an additive effect against *B. bigemina*, *B. caballi* and *T. equi.* Combination treatments of APSP EA-DA showed an additive effect against *B. caballi*, *B. bovis* and *T. equi*, but a synergistic effect against *B. bigemina* and *B. divergens.* Combination treatments of APSP EA-AQ showed an additive effect against *B. caballi*, while a synergistic effect against *B. bovis*, *B. bigemina*, *B. divergens* and *T. equi*. Combination treatments of APSP EA-CF showed an additive effect against all tested parasites; none of the combinations showed an antagonistic effect (Table 2).

**Table 2:** The effect of combinations of EA, β-CD EA and APSP EA with DA, AQ and CF against *Babesia* and *Theileria* parasites *in vitro*

Drug combination		*B. bovis*	*B. bigemina*	*B. divergens*	*B. caballi*	*T. equi*
EA + DA	CI value	0.402	1.046	0.317	0.854	0.474
	Interaction	Synergism	Additive	Synergism	Synergism	Synergism
β-CD EA + DA	CI value	1.003	0.953	1.004	0.830	0.970
	Interaction	Additive	Additive	Additive	Synergism	Additive
APSP EA + DA	CI value	0.965	0.832	0.527	1.003	0.908
	Interaction	Additive	Synergism	Synergism	Additive	Additive
EA + AQ	CI value	0.005	0.225	0.318	0.153	0.352
	Interaction	Synergism	Synergism	Synergism	Synergism	Synergism
β-CD EA + AQ	CI value	0.027	0.004	0.103	0.539	0.809
	Interaction	Synergism	Synergism	Synergism	Synergism	Synergism
APSP EA + AQ	CI value	0.609	0.801	0.325	0.902	0.780
	Interaction	Synergism	Synergism	Synergism	Additive	Synergism
EA + CF	CI value	0.788	0.817	1.036	1.079	1.100
	Interaction	Synergism	Synergism	Additive	Additive	Additive
β-CD EA + CF	CI value	0.032	1.001	0.002	0.900	1.003
	Interaction	Synergism	Additive	Synergism	Additive	Additive
APSP EA + CF	CI value	1.001	0.907	1.005	1.099	1.000
	Interaction	Additive	Additive	Additive	Additive	Additive

*Abbreviations*: DA, diminazene aceturate; AQ, atovaquone; CF, clofazimine; EA, ellagic acid; β-CD-EA, β-cyclodextrin ellagic acid; APSP EA, antisolvent precipitation with syringe pump prepared ellagic acid; CI, combination index

### Toxicity of EA, β-CD EA, APSP EA, DA, AQ and CF on MDBK, NIH/3T3 and HFF cell lines

EA, β-CD EA and APSP EA showed effective inhibition on the *in vitro* culture of *Babesia* and *Theileria* parasites similar to that with CF. Therefore, the effect of EA, β-CD EA and APSP EA on the host cells was evaluated using MDBK, NIH/3T3 and HFF cell lines to see the cytotoxicity of these compounds (Table 1). EA and β-CD EA at concentrations of 800 µM did not show any inhibition of MDBK and NIH/3T3 cell viability, while APSP EA showed inhibition only on HFF with an EC_50_ value of 790 ± 5.4 µM (Table 1). In a separate assay, DA and AQ at concentrations of 100 µM did not show any inhibition of MDBK, NIH/3T3 and HFF cell viability, while CF showed inhibition only on MDBK with an EC_50_ value of 34 ± 3.4 µM (Additional file 2: Table S1). Selectivity indexes are defined as the ratio of EC_50_ of the tested compounds of the cell line to the IC_50_ of these compounds on the parasite. For EA, the highest selectivity index was achieved on *B. caballi*; in the case of MDBK, NIH/3T3 and HFF cell lines, it was found to be 22.7 times higher than the IC_50_. For β-CD EA and APSP EA, the highest selectivity index was achieved on *B. caballi*; in the case of MDBK, NIH/3T3 and HFF cell lines, it was found to be 22.7 and 111.1 times higher than its IC_50_, respectively (Table 1).

### The chemotherapeutic effect of EA, β-CD EA and APSP EA against *B. microti* in mice

**Fig. 4:**
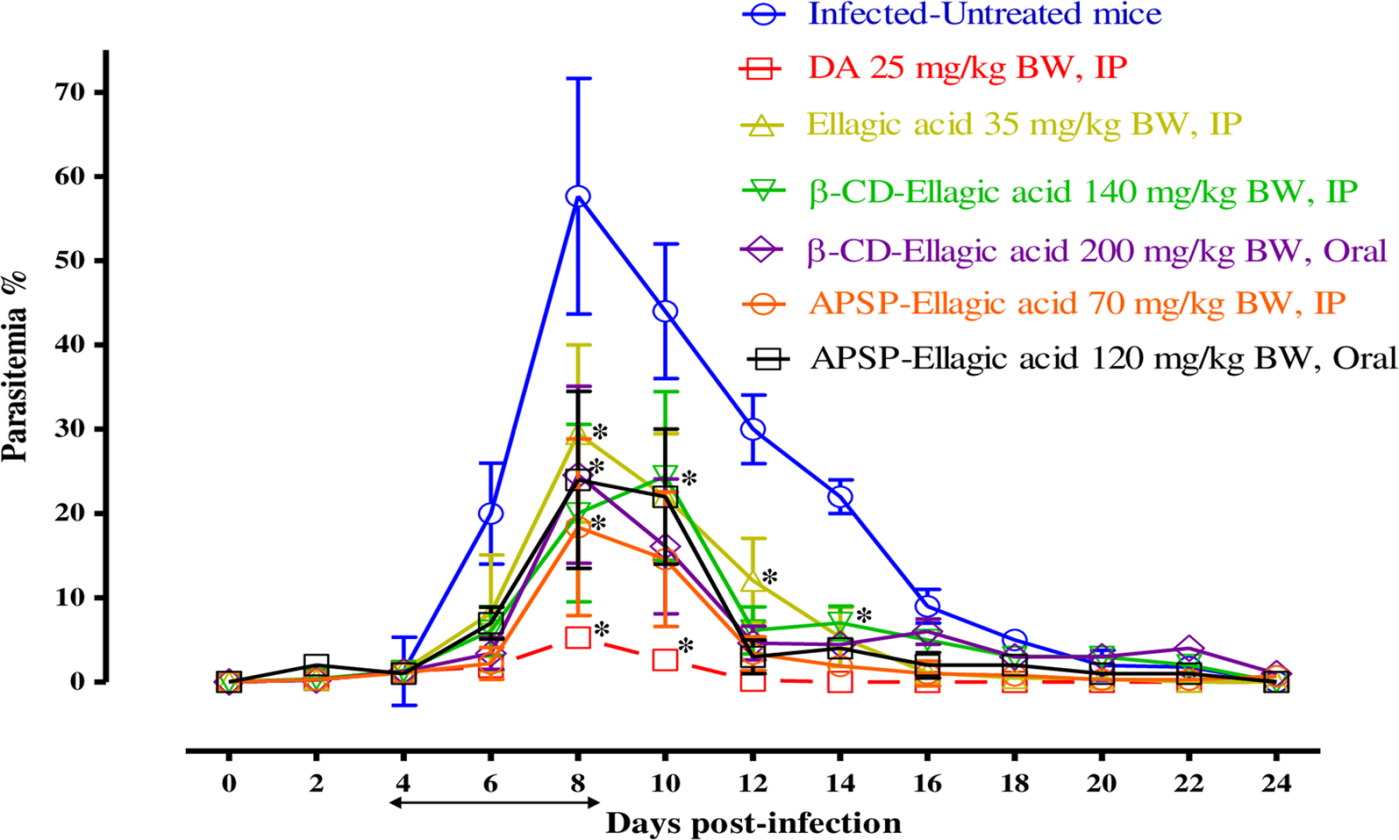
Growth inhibition of EA, β-CD EA and APSP EA on *B. microti in vivo.* Graph showing the inhibitory effects of DA-IP, EA-IP, β-CD EA-IP, β-CD EA-oral, APSP EA-IP and APSP EA-oral treatment compared to the untreated group. The values plotted indicate the mean ± standard deviation for two separate experiments. Asterisks (*) indicate statistically significant differences (*P* < 0.05) based on the unpaired t-test analysis. The arrow indicates 5 consecutive days of treatment. Parasitemia was calculated by counting infected RBCs among 5000 RBCs using Giemsa-stained thin blood smears. *Abbreviations*: DA, diminazene aceturate; EA, ellagic acid; β-CD EA, β-cyclodextrin ellagic acid; APSP EA, antisolvent precipitation with syringe pump prepared ellagic acid; IP, intraperitoneal; RBCs, red blood cells

**Fig. 5:**
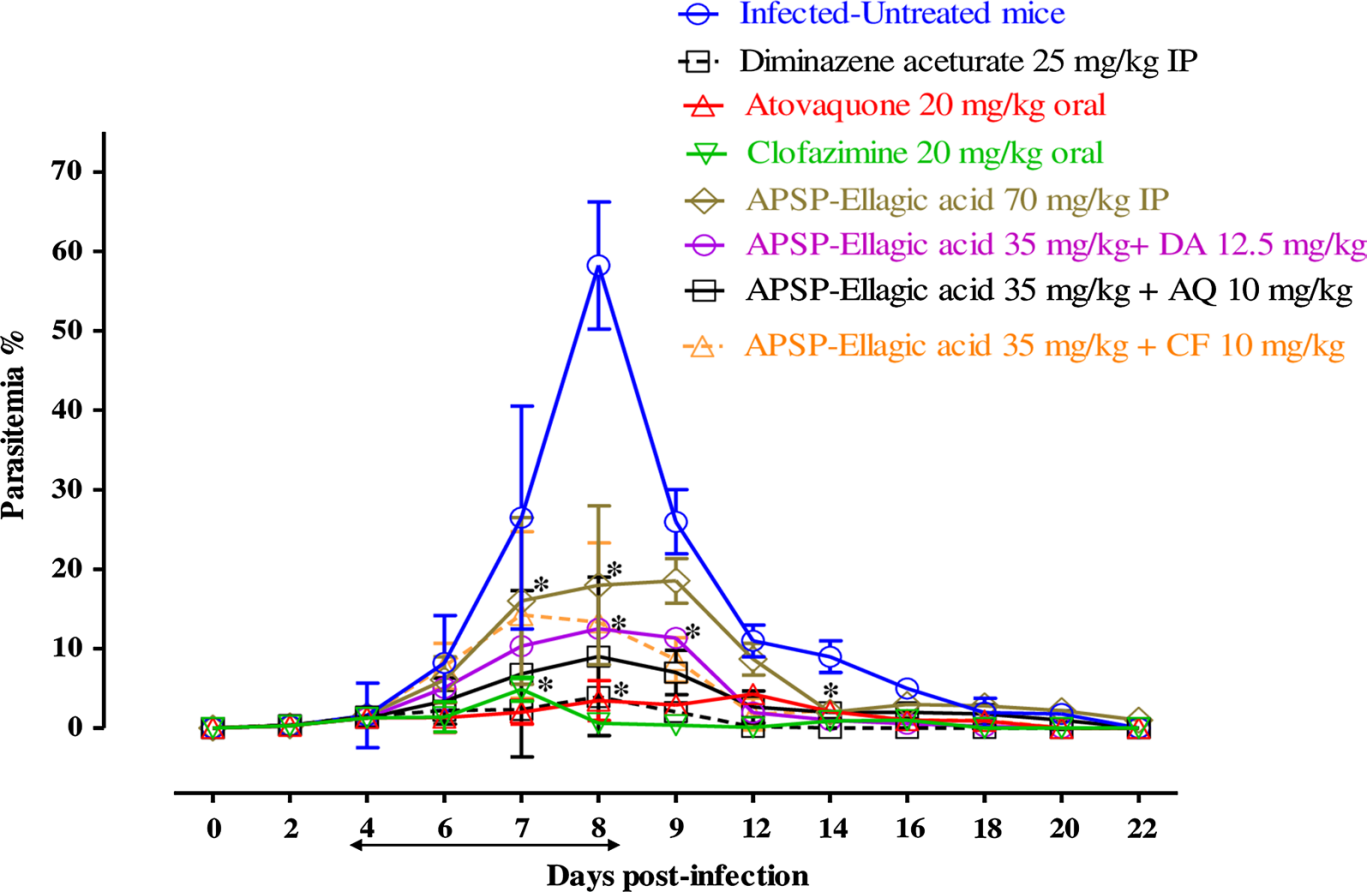
Growth inhibition of APSP-ellagic acid on *B. microti in vivo.* Graph showing the inhibitory effects of DA-IP, AQ-oral, CF-oral, APSP EA-IP, APSP EA-DA, APSP EA-AQ and APSP EA-CF treatment as compared to the untreated group. The values plotted indicate the mean ± standard deviation for two separate experiments. Asterisks (*) indicate statistically significant differences (*P* < 0.05) based on the unpaired t-test analysis. The arrow indicates 5 consecutive days of treatment. Parasitemia was calculated by counting infected RBCs among 5000 RBCs using Giemsa-stained thin blood smears. *Abbreviations*: DA, diminazene aceturate; AQ, atovaquone; CF, clofazimine; EA, ellagic acid; APSP EA, antisolvent precipitation with syringe pump prepared ellagic acid; IP, intraperitoneal; RBCs, red blood cells

**Fig. 6:**
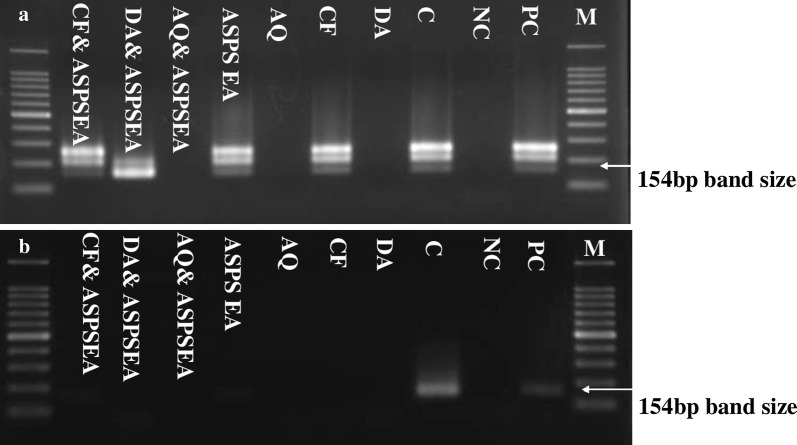
Molecular detection of parasite DNA in the blood of treated groups. Images **a**, **b** show the molecular detection of parasites in the blood of treated groups. Double distilled water (DDW) was used as a negative control. The arrow shows the expected band length of 154 bp for positive cases of *B. microti. Abbreviations*: PC, positive control; NC, negative control; M, marker; C, *B. microti* DNA; DA, diminazene aceturate; AQ, atovaquone; CF, clofazimine; EA, ellagic acid; APSP EA, antisolvent precipitation with syringe pump prepared ellagic acid; β-CD EA, β-cyclodextrin ellagic acid

To further evaluate the efficacies of EA, β-CD EA and APSP EA as compared with those of other drugs, the chemotherapeutic effects of EA, β-CD EA and APSP EA were examined in mice infected with *B. microti* (Fig. 4). In the first assay to detect whether EA or its loaded nanoparticles are more effective, in the DDW control group, the multiplication of *B. microti* increased significantly and reached the highest parasitemia at 57.7% on day 8 post-infection (p.i.). In all treated groups, the level of parasitemia was cleared at a significantly lower percentage of parasitemia than that of the control group (t-test: *t*_(12)_ = 3.39, *P* = 0.005 for EA-treated; *t*_(12)_ = 2.739, *P* = 0.001 for β-CD EA i.p.-treated and β-CD EA oral-treated; *t*_(12)_ = 3.17, *P* = 0.007 for APSP EA i.p.-treated and APSP EA oral-treated groups) from day 6 to 12 p.i. In all treated mice, the peak parasitemia level reached 29.5% on day 8, 24% on day 10, 24% on day 8, 18.4% on day 8, 24% on day 8 and 5.2% on day 8 in 35 mg/kg EA i.p., 140 mg/kg β-CD EA i.p., 200 mg/kg β-CD EA oral, 70 mg/kg APSP EA i.p., 120 mg/kg APSP EA oral and 25 mg/kg DA i.p., respectively (Fig. 4). The hematocrit (HCT) count of the DDW control group was determined to be significantly different from the counts of all drug-treated groups (t-test: *t*_(3)_ = 1.15, *P* = 0.01 for EA-treated; *t*_(3)_ = 4.380, *P* = 0.02 for β-CD EA i.p.-treated and β-CD EA oral-treated; *t*_(3)_ = 2.298, *P* = 0.001 for APSP EA i.p.-treated and APSP EA oral-treated groups) (Additional file 3: Figure S2). In the second assay, we continued with APSP EA in the DDW control group, where the multiplication of *B. microti* increased significantly and reached the highest parasitemia at 58.3% on day 8 p.i. (t-test: *t*_(12)_ = 2.782, *P* < 0.0001 for APSP EA-treated; *t*_(12)_ = 2.528, *P* < 0.0001 for APSP EA-DA-treated, APSP EA-AQ-treated and APSP EA-CF-treated groups). In the mono-chemotherapy-treated mice, the peak parasitemia level reached 18.6, 3.9, 4.3 and 4.9% in 70 mg/kg APSP EA, 25 mg/kg DA, 20 mg/kg AQ and 20 mg/kg CF, respectively (Fig. 5). Parasitemia was undetectable in mice treated with 25 mg/kg DA, 20 mg/kg AQ and 20 mg/kg CF by microscopic examination starting on day 13, 15 and 16 p.i., respectively. On day 26 p.i., parasitemia was undetectable by microscopic examination in mice treated with 70 mg/kg of APSP EA. Meanwhile, in groups treated with combination chemotherapy, the peak parasitemia levels reached 12.5, 14.2 and 9% in 35 mg/kg APSP EA-12.5 mg/kg DA, 35 mg/kg APSP EA-10 mg/kg CF and 35 mg/kg APSP EA-10 mg/kg AQ, respectively, on day 9 (Fig. 5). Parasitemia was undetectable in mice by microscopic examination on days 18, 22 and 18 p.i. with (35 mg/kg APSP EA-12.5 mg/kg DA), (35 mg/kg APSP EA-10 mg/kg CF) and (35 mg/kg APSP EA-10 mg/kg AQ), respectively. Furthermore, infection with *B. microti* reduces the RBCs, HGB concentration and HCT percentage in mouse blood, as observed in the DDW control group on days 8 and 12 p.i. The RBCs (t-test: *t*_(6)_ = 4.367, *P* = 0.005 for APSP EA-treated; *t*_(6)_ = 4.756, *P* =0.003 for APSP EA-DA-treated, APSP EA-AQ-treated and APSP EA-CF-treated groups) (Additional file 4: Figure S3a), HGB concentration (t-test: *t*_(6)_ = 4.25, *P* = 0.005 for APSP EA-treated; *t*_(6)_ = 5.736, *P* < 0.0001 for APSP EA-DA-treated, APSP EA-AQ-treated and APSP EA-CF-treated groups) (Additional file 4: Figure S3b) and HCT percentage (t-test: *t*_(6)_ = 7.768, *P* = 0.0002 for APSP EA-treated; *t*_(6)_ = 8.303, *P* < 0.0001 for APSP EA-DA-treated, APSP EA-AQ-treated and APSP EA-CF-treated groups) (Additional file 4: Figure S3c) were also determined to be significantly different between the DDW control group and all drug-treated groups. Parasite DNA was not detected in (25 mg/kg DA), (20 mg/kg AQ) and (35 mg/kg APSP EA-10 mg/kg AQ) on day 42 (Fig. 6a). Meanwhile, on day 56, parasite DNA was not detected in any group (Fig. 6b).

## Discussion

Diminazene aceturate (DA) and imidocarb dipropionate are still the drug options for treating bovine and equine piroplasmosis, while clindamycin–quinine and atovaquone–azithromycin combinations are still used for human babesiosis management [31, 32]. Recently, the resistance of piroplasms against the current drug molecules, the toxic effects after treatment and the drug residue inside animal tissues have been documented [33]. Therefore, there is an urgent need to discover new potent compounds that will provide more options for human babesiosis as well as equine and bovine piroplasmosis treatment [34].

In the present study, the absorption spectral data and SEM analysis of nanoparticles confirmed their unique and distinctive characteristics, such as the ability to improve the solubility ratio and a large surface area-to-volume ratio that can be explored for biomedical purposes [15, 20, 30]. EA and its microspheres (β-CD EA and APSP EA) were effective against *Babesia* and *Theileria* parasites *in vitro*, while APSP EA showed the lowest IC_50_ values. Previous reports documented EA’s strong inhibition of the growth of many protozoan parasites, including *Plasmodium*, *Trypanosoma* and *Leishmania*, by different action mechanisms [12–14]. However, the mode of action is yet to be understood comprehensively in comparison to the existing data. Aminu et al. [13] showed that the trypanosome-suppressive effect of EA was attributed to its polyphenolic nature and antioxidant activity that could largely prevent trypanosome-associated oxidative stress and spare the organs from oxidative damage, whereas Alves et al. [14] showed that EA reduced the number of infected macrophages and the survival index of *Leishmania major* internalized amastigotes in parasitized macrophages, as well as induced macrophage activation by increasing the phagocytic capability, lysosomal volume, NO synthesis and cytoplasmic calcium release. Soh et al. [12] reported that EA can have both antioxidant and antiplasmodial properties, and the potential antioxidant action of EA against *P. falciparum* has no effect on its high *in vitro* antiplasmodial activity. Glutathione (GSH) plays a role in the antimalarial properties of EA, whereas EA inhibited the growth of *Plasmodium* by reducing the GSH content inside the parasite. Regarding the close biological similarities between *Plasmodium*, *Babesia* and *Theileria* parasites, we suggest that EA may act by the same pathway against *Babesia* and *Theileria* parasites. However, it is imperative that further studies are conducted to confirm the exact mechanism of action of EA and its microspheres against *Babesia* and *Theileria.*

Combination chemotherapy has been recommended to enhance the potency of drugs by reducing their dosage, leading to a reduction in toxic side effects as well as drug-resistant parasites. The effectiveness of EA, β-CD EA and APSP EA were further evaluated in a combination study using DA, AQ and CF *in vitro*. Synergetic and additive effects were found between EA, β-CD EA or APSP EA and current antibabesial drugs (DA, AQ, CF), which is consistent with the findings of Soh et al. [7], who revealed that EA showed synergistic activity with current antimalarial drugs (chloroquine, artesunate, mefloquine and atovaquone). Therefore, EA or its microspheres and DA combinations might be used as a novel regime for treating piroplasmosis in a wide range of animals. Furthermore, EA or its microspheres and AQ or CF combinations could be used as an option for treating human babesiosis.

The safety of drug therapy is one of the most important tasks of modern health care. Recently, animal cell cultures are increasingly found in toxicological studies methods as an alternative to the classical experimental animal tests [35]. Additionally, these methods can significantly solve the ethical problems associated with the mass use and death of experimental animals, and reduce the cost and time of preliminary studies of new chemical drugs mainly at the stage of pre-clinical trials. These test cultures of human or animal cells are derived from different tissues and organs of different sensitivities to chemicals [36]. Therefore, the cytotoxicity test was performed using three different types of culture cell lines to determine the effect of EA and its microspheres on the bovine, mouse and human cells which are the main hosts of *Babesia* and *Theileria* parasites. The toxicity assay showed that EA, β-CD EA and APSP EA did not affect the viability of MDBK, NIH/3T3 and HFF cell lines. This finding is consistent with the results reported by Losso et al. [37], who showed that EA did not affect the viability of normal fibroblast cells. Recently, Weisburg et al. [38] explained that EA was cytotoxic to carcinoma cells without affecting normal cells. This suggests that EA might be safe for use in humans and animals following further *in vivo* clinical studies.

The potent effect of EA, β-CD EA and APSP EA, as well as the additive and synergistic effects in combinations *in vitro*, encouraged us to further evaluate their *in vivo* effects on *B. microti* in a mouse model. APSP EA administered intraperitoneally showed higher chemotherapeutic effects than EA and β-CD EA against *B. microti* without any toxic symptoms in mice. Interestingly, the combination treatment of APSP EA and AQ at a half dose showed a potent chemotherapeutic effect comparable to that of those single drugs at full dose, emphasizing that APSP EA is a good combinatorial drug. Regarding the chemotherapeutic effects of EA against *Plasmodium* parasites in mice, Soh et al. [7] reported that intraperitoneal administration of EA to *P. vinckei petteri*-infected mice showed high curative and prophylactic effects without any toxic effect. Interestingly, β-CD EA postponed peak parasitemia in mice when administered i.p. from day 8 to day 10, which indicates that EA was enclosed effectively by β-CD to form EA microspheres. These results are compatible with those of previous studies [16, 19]. Interestingly, Wang et al. [16] revealed that the inclusion of EA with microspheres enhanced its stability and also conferred it with more functions and excellent properties. Therefore, cyclodextrin could be a useful delivery system for improving the bioavailability and therapeutic efficiency of EA and other poorly water-soluble drugs to treat piroplasmosis [19]. Hematology profiles (RBCs, HGB and HCT count) and parasitemia were also improved in comparison with the DDW control group.

A PCR assay was performed on blood samples collected on days 42 and 56 post-infection to analyze the presence of *B. microti* DNA. Interestingly, this study confirmed the absence of *B. microti* DNA in groups treated with DA, AQ and combination chemotherapy of APSP EA-AQ as compared to monotherapy on day 42. On day 56, parasite DNA was not detected in any of the groups. These results confirm the ability of APSP EA to eliminate *B. microti* in mice and the importance of combination chemotherapy for the effective control of piroplasmosis [39]. However, it is very difficult to determine whether these effects are due to the inhibition of growth or death of parasites since each drug has a different mode of action and one action might affect the other of a different drug. Furthermore, for *in vivo* chemotherapeutic experiments, these effects may be also due to the host immune response to the infection. Taken together, these findings advocate that APSP EA is a potential drug against bovine babesiosis, equine piroplasmosis and human babesiosis.

## Conclusions

EA, β-CD EA and APSP EA showed growth-inhibitory effects against several *Babesia* species and *T. equi in vitro* and chemotherapeutic efficacy against *B. microti in vivo*. However, we did not perform a viability test in order to assess the parasite viability as reported by AbouLaila et al. [40], who documented that all tested parasites could not revive even after drug withdrawal. Therefore, a viability assay is required to detect whether the effect of EA and EA-NPs is inhibition of the growth or death in the future study. The effectiveness of APSP EA *in vivo* was comparable to that shown by DA in mice. The APSP EA-AQ combination showed higher efficiency against *B. microti* in mice than did APSP EA monotherapy. β-CD EA microspheres were successfully prepared and postponed the peak parasitemia on *B. microti*-infected mice by slow the release of EA from the forming microspheres. However, further experiments are needed to evaluate the side effects of EA-NPs on the histopathological and biochemical changes in different tissues and different times of treated mice. Conclusively, EA-loaded nanoparticles are a promising route for promoting EA bioavailability and solubility, while improving/sustaining its antibabesial efficacy *in vitro* and *in vivo*.

## Additional files

**Additional file 1: Figure S1.** **a** The correlation between RFUs and the log concentrations of EA (nM) on *Babesia* and *Theileria* parasites. **b** The correlation between RFUs and the log concentrations of APSP EA (nM) on *Babesia* and *Theileria* parasites. **c** The correlation between RFUs and the log concentrations of β-CD EA (nM) on *Babesia* and *Theileria* parasites. The values plotted were obtained from three separate trials of the fluorescence assay, using the non-linear regression (curve fit analysis) in GraphPad Prism software. Asterisks (*) indicate the drug concentration that significantly (*P* < 0.05) inhibited the growth of all tested species. *Abbreviations*: EA, ellagic acid; β-CD EA, β-cyclodextrin ellagic acid; APSP EA, antisolvent precipitation with syringe pump prepared ellagic acid; RFUs, relative fluorescence units.

**Additional file 2: Table S1.** The IC_50_ and selectivity index of DA, AQ and CF.

**Additional file 3: Figure S2.** Hematocrit (HCT) changes in EA-treated, β-CD EA-treated and APSP EA-treated mice *in vivo* as compared with untreated mice. The values plotted are the mean ± standard deviation for two separate trials. Asterisks (*) indicate statistical significance (*P* < 0.05) based on unpaired t-test analysis. *Abbreviations*: DA, diminazene aceturate; IP, intraperitoneal; BW, body weight.

**Additional file 4: Figure S3.** Changes in red blood cell (RBC) (**a)**, hemoglobin (HGB) (**b)** and hematocrit (HCT) (**c**) values in APSP EA-treated mice *in vivo*. The values plotted are the mean ± standard deviation for two separate trials. Asterisks (*) indicate statistical significance (*P* < 0.05) based on the unpaired t-test analysis. The arrow indicates 5 consecutive days of treatment. *Abbreviations*: DA, diminazene aceturate; AQ, atovaquone; CF, clofazimine; EA, ellagic acid; APSP EA, antisolvent precipitation with syringe pump prepared ellagic acid; IP, intraperitoneal.

## Data Availability

Data supporting the conclusions of this article are included within the article and its additional files. The datasets generated and/or analyzed during the present study are available from the corresponding author upon reasonable request.

## Abbreviations

EA: ellagic acid
EA-NPs: EA-loaded nanoparticles
DA: diminazene aceturate
AQ: atovaquone
CF: clofazimine
β-CD EA: β-cyclodextrin ellagic acid
APSP EA: antisolvent precipitation with a syringe pump prepared ellagic acid
DMSO: dimethyl sulfoxide
MDBK: Madin-Darby bovine kidney
NIH/3T3: mouse embryonic fibroblast
HFF: human foreskin fibroblast
RBCs: red blood cells
SEM: scanning electron microscope
CCK-8: Cell Counting Kit-8
iRBCs: infected red blood cells
DDW: double-distilled water
BW: body weight
HGB: hemoglobin
HCT: hematocrit
PCR: polymerase chain reaction
IC_50_: half-maximal inhibitory concentration
EC_50_: half-maximal effective concentration
GSH: glutathione
