# Supplementary figures and images for: Ellagic acid microspheres restrict the growth of Babesia and Theileria in vitro and Babesia microti in vivo
Source: Parasit Vectors. 2019 May 28;12:269. doi: 10.1186/s13071-019-3520-x (PMC6537213; doi:10.1186/s13071-019-3520-x)

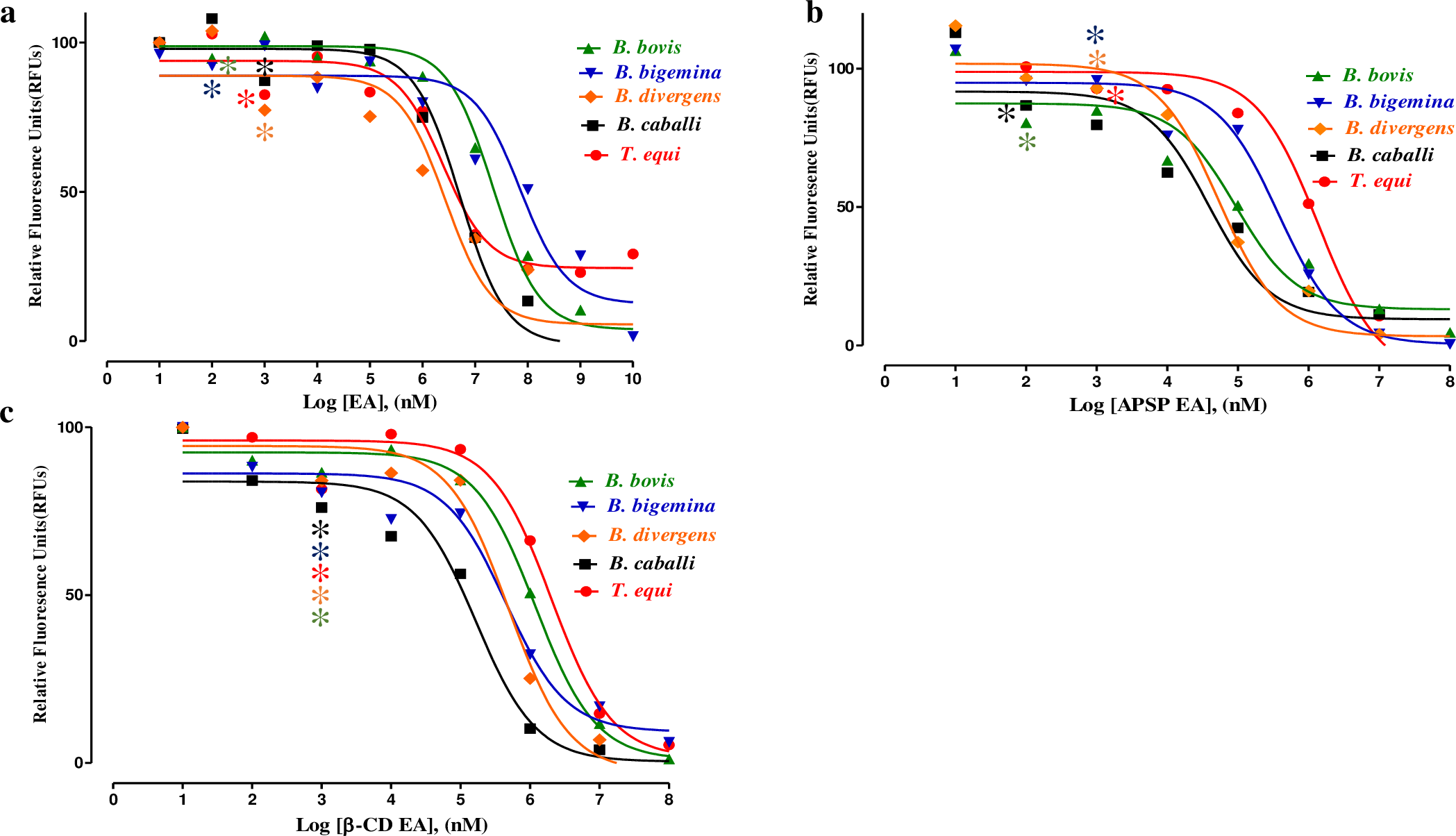

Supplement: Supplementary file 1 — Additional file 1: Figure S1. a The correlation between RFUs and the log concentrations of EA (nM) on Babesia and Theileria parasites. b The correlation between RFUs and the log concentrations of APSP EA (nM) on Babesia and Theileria parasites. c The correlation between RFUs and the log concentrations of β-CD EA (nM) on Babesia and Theileria parasites. The values plotted were obtained from three separate trials of the fluorescence assay, using the non-linear regression (curve fit analysis) in GraphPad Prism software. Asterisks (*) indicate the drug concentration that significantly (P < 0.05) inhibited the growth of all tested species. Abbreviations: EA, ellagic acid; β-CD EA, β-cyclodextrin ellagic acid; APSP EA, antisolvent precipitation with syringe pump prepared ellagic acid; RFUs, relative fluorescence units. [file 13071_2019_3520_MOESM1_ESM.tif]

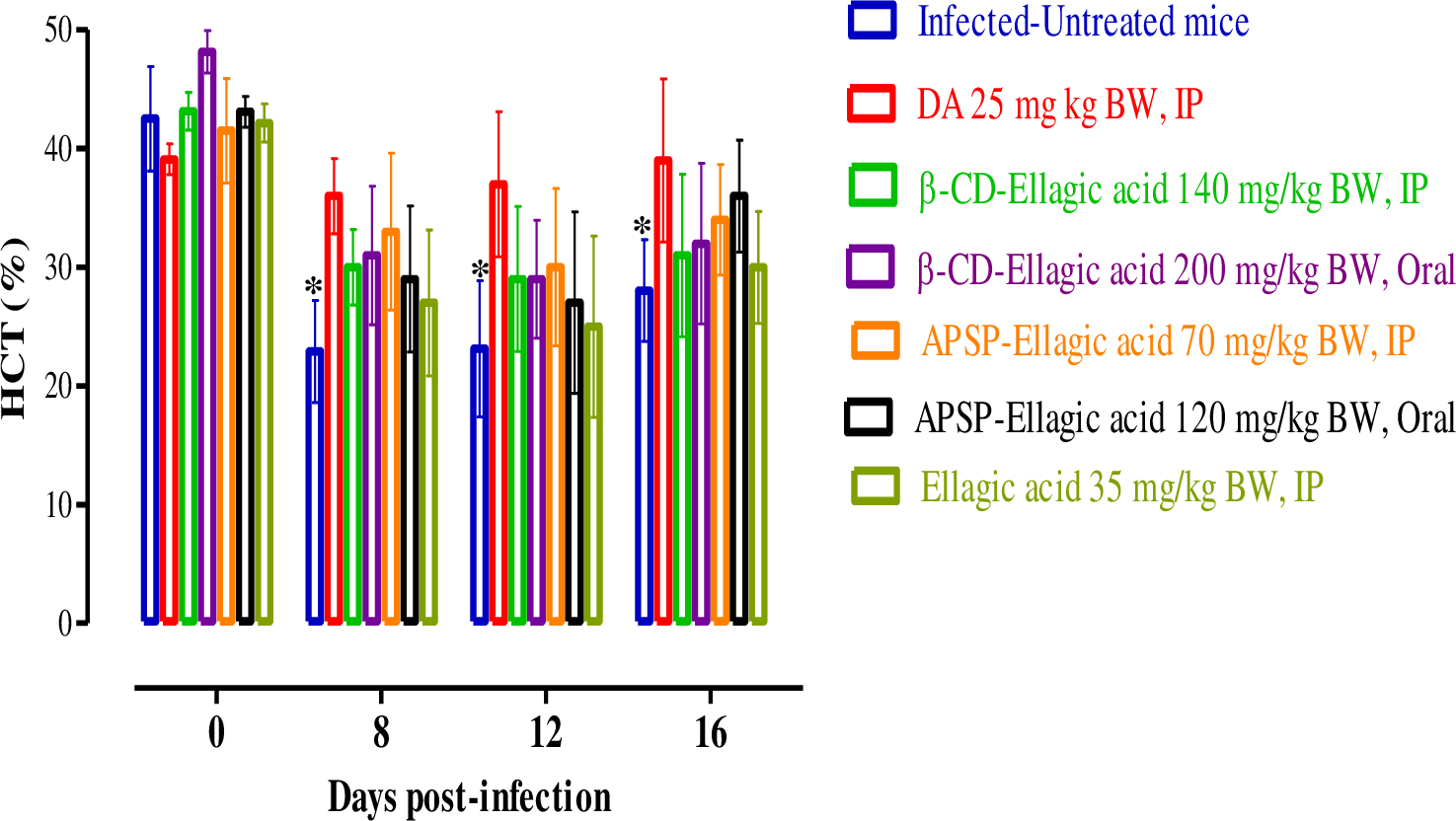

Supplement: Supplementary file 3 — Additional file 3: Figure S2. Hematocrit (HCT) changes in EA-treated, β-CD EA-treated and APSP EA-treated mice in vivo as compared with untreated mice. The values plotted are the mean ± standard deviation for two separate trials. Asterisks (*) indicate statistical significance (P < 0.05) based on unpaired t-test analysis. Abbreviations: DA, diminazene aceturate; IP, intraperitoneal; BW, body weight. [file 13071_2019_3520_MOESM3_ESM.tif]

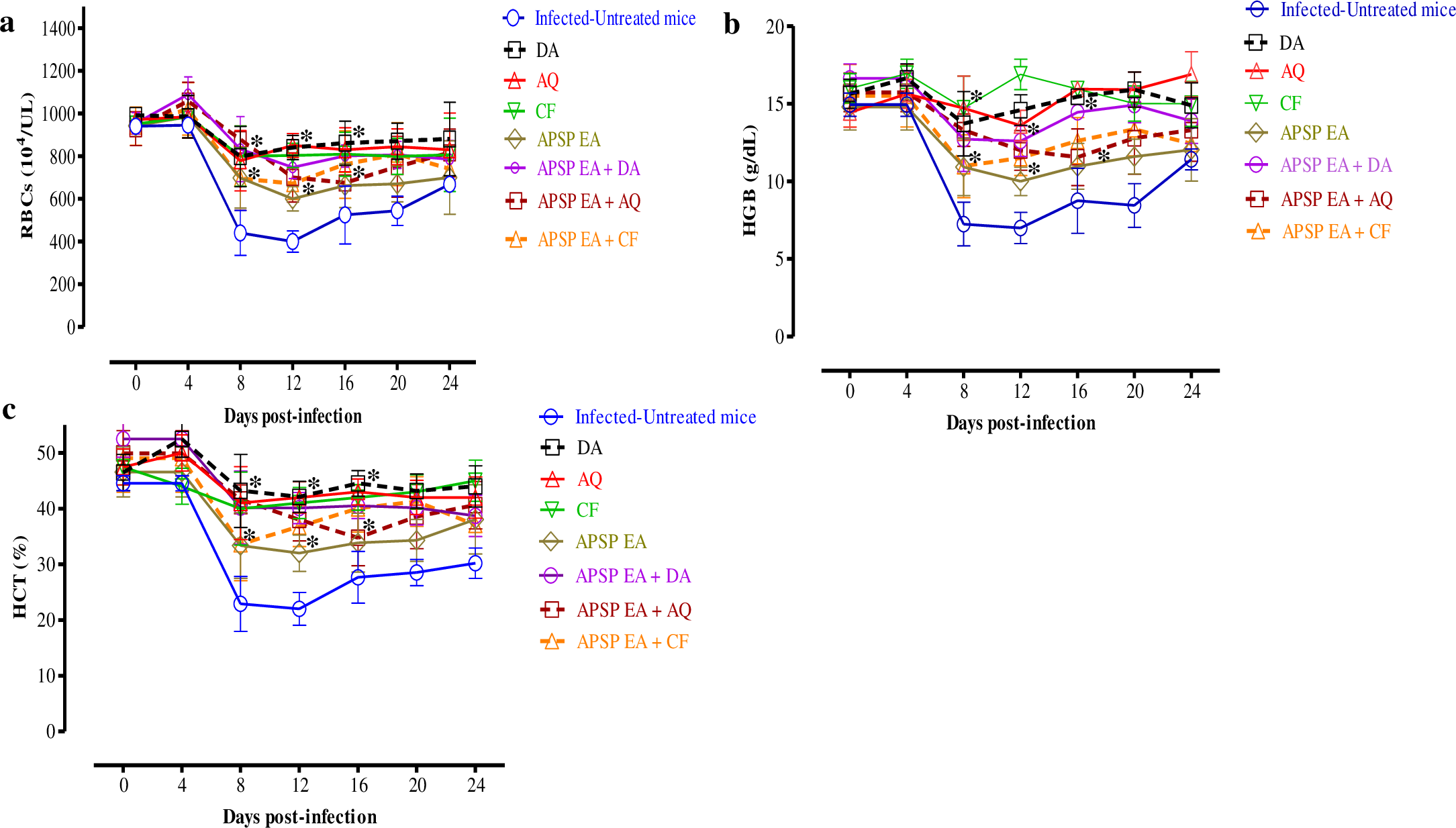

Supplement: Supplementary file 4 — Additional file 4: Figure S3. Changes in red blood cell (RBC) (a), hemoglobin (HGB) (b) and hematocrit (HCT) (c) values in APSP EA-treated mice in vivo. The values plotted are the mean ± standard deviation for two separate trials. Asterisks (*) indicate statistical significance (P < 0.05) based on the unpaired t-test analysis. The arrow indicates 5 consecutive days of treatment. Abbreviations: DA, diminazene aceturate; AQ, atovaquone; CF, clofazimine; EA, ellagic acid; APSP EA, antisolvent precipitation with syringe pump prepared ellagic acid; IP, intraperitoneal. [file 13071_2019_3520_MOESM4_ESM.tif]
