# Supplementary material for: Ellagic acid microspheres restrict the growth of Babesia and Theileria in vitro and Babesia microti in vivo
Source: Parasit Vectors. 2019 May 28;12:269. doi: 10.1186/s13071-019-3520-x (PMC6537213; doi:10.1186/s13071-019-3520-x)
Supplement: Supplementary file 2 — Additional file 2: Table S1. The IC50 and selectivity index of DA, AQ and CF. [file 13071_2019_3520_MOESM2_ESM.docx]

**Additional file 2: Table S1** The IC_50_ and selectivity index of DA, AQ, and CF

| **Compound** | ***Babesia* and *Theileria*** | **IC_50_ (µM)^a^** | **EC_50_ (µM)^b^** | | | **Selective index^c^** | | |
| --- | --- | --- | --- | --- | --- | --- | --- | --- |
|  |  |  | **MDBK** | **NIH/3T3** | **HFF** | **MDBK** | **NIH/3T3** | **HFF** |
| **DA** | *B. bovis* | **0.35 ± 0.06** | **˃100** | **˃100** | **˃100** | **˃ 285.7** | **˃ 285.7** | **˃ 285.7** |
|  | *B. bigemina* | **0.68 ± 0.09** |  |  |  | **˃ 208.3** | **˃ 208.3** | **˃ 208.3** |
|  | *B. divergens* | **0.43 ± 0.05** |  |  |  | **˃ 232.5** | **˃ 232.5** | **˃ 232.5** |
|  | *B. caballi* | **0.02 ± 0.0002** |  |  |  | **˃ 4545** | **˃ 4545** | **˃ 4545** |
|  | *T. equi* | **0.71 ± 0.05** |  |  |  | **˃ 476.2** | **˃ 476.2** | **˃ 476.2** |
| **AQ** | *B. bovis* | **0.039 ± 0.002** | **˃100** | **˃100** | **˃100** | **˃ 10.4** | **˃ 10.4** | **˃ 10.4** |
|  | *B. bigemina* | **0.701 ± 0.04** |  |  |  | **˃ 12.7** | **˃ 12.7** | **˃ 12.7** |
|  | *B. divergens* | **0.038 ± 0.002** |  |  |  | **˃ 18.5** | **˃ 18.5** | **˃ 18.5** |
|  | *B. caballi* | **0.102 ± 0.014** |  |  |  | **˃ 30.4** | **˃ 30.4** | **˃ 30.4** |
|  | *T. equi* | **0.095 ± 0.065** |  |  |  | **˃ 13.4** | **˃ 13.4** | **˃ 13.4** |
| **CF** | *B. bovis* | **8.24 ± 1.7** | **34.7 ± 3.4** | **˃100** | **˃100** | **4.2** | **˃ 12.1** | **˃ 12.1** |
|  | *B. bigemina* | **5.73 ± 1.9** |  |  |  | **6.1** | **˃ 17.5** | **˃ 17.5** |
|  | *B. divergens* | **13.85 ± 4.3** |  |  |  | **2.5** | **˃ 7.2** | **˃ 7.2** |
|  | *B. caballi* | **7.95 ± 1.8** |  |  |  | **4.4** | **˃ 12.6** | **˃ 12.6** |
|  | *T. equi* | **2.88 ± 0.9** |  |  |  | **12.1** | **˃ 34.7** | **˃ 34.7** |

^a^Half-maximal inhibition concentration of DA, AQ, and CF on the *in vitro* culture of parasites. The value was determined from the dose-response curve using nonlinear regression (curve fit analysis). The values are the means of experiments run in triplicate

^b^Half-maximal effective concentration of AQ, DA, and CF on cell lines. The values were determined from the dose-response curve using nonlinear regression (curve fit analysis). The values are the means of experiments in triplicate

^c^Ratio of the EC_50_ of cell lines to the IC_50_ of each species. High numbers are favorable

*Abbreviations:* *DA*, diminazene aceturate; *AQ*, atovaquone; *CF*, clofazimine; *MDBK*, Madin–Darby bovine kidney; *NIH/3T3*, Mouse embryonic fibroblast; *HFF*, Human foreskin fibroblast
